# Selective transfer of perceptual learning in parafoveal second-order motion: generalization to first-order motion but not to static second-order stimuli

**DOI:** 10.3389/fnins.2026.1953237

**Published:** 2026-09-18

**Authors:** Yong Tang, Yifeng Zhou

**Affiliations:** 1 Department of Medical Technology, Anhui Institute of Medicine, Hefei, China; 2 Anhui Provincial Center for Maternal and Child Health Genetics, Hefei, China; 3 Hefei National Laboratory for Physical Science at the Microscale, Division of Life Sciences and Medicine, University of Science and Technology of China, Hefei, China; 4 State Key Laboratory of Brain and Cognitive Science, Institute of Psychology, Chinese Academy of Sciences, Hefei, China

**Keywords:** first-order motion, motion perception, perceptual learning, second-order motion, transfer of learning

## Abstract

Perceptual learning of motion provides a powerful tool for investigating the hierarchical organization of the visual system. Previous studies have shown that training on second-order (contrast-defined) motion transfers asymmetrically to first-order (luminance-defined) motion, but the locus of this learning effect remains unclear. Here we tested whether second-order motion training transfers to static second-order perception, a condition that shares early texture-defined features but lacks motion components. Twenty-four subjects were assigned to a training group (*n* = 16) or a control group (*n* = 8). The training group underwent eight sessions of second-order motion direction discrimination training in the parafovea, while the control group received no training. Contrast sensitivity for second-order motion, first-order motion, and static first- and second-order stimuli was measured before and after training. The training group showed substantial improvements in contrast sensitivity for both second-order and first-order motion, with gains of approximately 1.8 dB and 2.7 dB, respectively. These improvements were significantly greater than those in the control group for motion stimuli. In contrast, improvements in static second-order perception were comparable between the training and control groups, indicating no specific training effect. Correlation analyses revealed a significant relationship between improvements in first- and second-order motion, but no correlation between second-order motion and static second-order perception. These findings suggest that second-order motion training primarily strengthens processing at a stage dedicated to motion extraction, rather than at early texture-feature extraction stages, providing behavioral evidence for the locus of plasticity within the dual-pathway model.

## Introduction

First- and second-order stimuli are two types of stimuli that are defined by different types of physical cues. The former is defined by luminance cues and is detected by simple energy-based mechanisms in V1, while the latter is defined by contrast or texture variations, and requires additional processing to extract the envelope (Cavanagh and Mather, 1989; Chubb and Sperling, 1989; Wilson et al., 1992; Lu and Sperling, 2001). The standard filter-rectify-filter (FRF) model captures the early distinction: first-order signals pass through orientation- and frequency-selective linear filters, while second-order signals require a nonlinear rectification step and a second filtering stage involving both cortical and subcortical contributions (Lu and Sperling, 2001; Ju et al., 2022). Despite this early distinction, what happens downstream remains less settled. One line of evidence points to convergence: first- and second-order signals may share a common motion-processing stage, presumably in V5 + (Albright, 1992; Dupont et al., 2003; Nishida et al., 2003; Seiffert et al., 2003; Hong et al., 2012; Bussieres and Casanova, 2017). Others find evidence for separate pathways, at both the local-motion-pooling level (Edwards and Metcalf, 2010) and the global-motion and optic-flow extraction levels (Badcock and Khuu, 2001; Ashida et al., 2007; Garcia-Suarez and Mullen, 2010; Cassanello et al., 2011; Vaina and Chubb, 2012). The relationship between the two systems, independent or converging, remains an open question.

Perceptual learning, improvements in performance with practice, offers a way to address this question. In the visual system, learning often exhibits specificity: improvements frequently fail to transfer across retinal locations, orientations, or spatial frequencies, suggesting involvement of early visual areas (Karni and Sagi, 1991; Ahissar and Hochstein, 1997). But the picture has become more nuanced. It appears that learning of complex or global features can generalize more readily, implying that higher-level areas are also involved (Gilbert et al., 2001; Ahissar and Hochstein, 2004). According to Lu and Dosher (2022), specificity is a graded phenomenon and serves as a powerful behavioral indicator of the cortical level involved in learning: transfer across stimulus features points to representations that have abandoned those properties, whereas specificity points to representations that preserve them. This logic, using transfer patterns to infer the locus of plasticity, provides a powerful tool for probing the motion processing hierarchy.

A striking asymmetry has emerged from previous studies: training on second-order motion substantially improves performance on first-order motion, but training on first-order motion does not transfer to second-order motion (Zanker, 1999; Chen et al., 2009; Petrov and Hayes, 2010). This asymmetric pattern rules out both pure independence (which would predict no cross-transfer) and full convergence (which would predict symmetric transfer), pointing instead to a more nuanced relationship, one that Petrov and Hayes (2010) sought to capture with their dual-pathway FRF model. In their model, a Fourier pathway responds only to luminance-defined signals, whereas a non-Fourier pathway responds to both luminance- and contrast-defined signals. Crucially, because the non-Fourier pathway processes both types of signals, it effectively embodies a convergence of first- and second-order motion information at the level of this pathway. A “MAX” stage then sits at the output of each task-relevant channel, comparing outputs from the two pathways and selecting the stronger one. The “MAX” stage also gates learning: only the pathway with the stronger response gets modified. During first-order training, the Fourier pathway dominates the MAX competition, so learning modifies only this pathway while the non-Fourier pathway remains unchanged, and transfer to second-order motion does not happen. During second-order training, the non-Fourier pathway is strengthened. Since this pathway responds to both luminance- and contrast-defined signals, the strengthened pathway can now support improved performance for both types of motion. The asymmetry in transfer thus arises from the asymmetric connectivity of the two pathways to this convergent MAX stage.

This account is plausible, but one key question remains unresolved. While the non-Fourier pathway embodies convergence of first- and second-order signals, the Petrov and Hayes model does not specify which stage within this pathway is strengthened by second-order motion training. In this model, the non-Fourier pathway spans multiple processing levels: early texture-feature extraction (layers b–d, including rectification and second filtering) and later motion extraction (Layer e, where direction-selective units first emerge). If training strengthens early texture-feature extraction, then improvements should transfer to any stimulus that shares these early texture-defined features, including static second-order stimuli. If training strengthens the motion extraction stage itself, then improvements should be confined to stimuli that engage motion processing and should not transfer to static stimuli that lack motion. To distinguish between these possibilities, we tested transfer to static second-order orientation discrimination, a task that shares early texture-defined features with second-order motion but lacks the motion component. If second-order motion training improved performance on this static task, that would suggest the training enhanced early texture-feature extraction (layers b–d). If it did not, that would point to a more specific locus: plasticity at the motion extraction stage (Layer e), where signals from both pathways converge onto common motion-sensitive units.

The above logic can be further formalized within the reweighting framework of Lu and Dosher (2022), where perceptual learning optimizes the connection weights between sensory representations and perceptual decisions without necessarily altering early representations themselves. In this framework, the extent of transfer depends on the degree of overlap and compatibility of connections for the training and transfer tasks. Applied to our case, this leads to a clear prediction: if second-order motion training strengthens connections at the motion extraction stage (Layer e), transfer should occur to first-order motion (which shares this stage) but not to static second-order perception (which does not engage this stage).

Notably, this critical prediction has not been tested in any retinal location: previous studies on asymmetric transfer (Zanker, 1999; Petrov and Hayes, 2010, for fovea; Chen et al., 2009, for parafovea) only assessed transfer to first-order motion, leaving the crucial static condition entirely untested. The parafovea offers a particularly informative testing ground for this prediction. Its reliance on coarser, more integrative mechanisms (Levi, 2008) is thought to constrain the potential loci of plasticity, thereby reducing contributions from early sensory stages and making it easier to isolate learning effects that arise from the later motion extraction stage (Layer e). Importantly, this logic rests on the assumption that the static task remains measurable in the parafovea; as long as baseline performance is well above chance, the absence of transfer would provide meaningful evidence against early-stage plasticity. To further rule out non-specific practice effects, we included a no-training control group, which was absent in prior work, allowing us to attribute any observed improvements specifically to training-induced plasticity.

To address the unresolved issues discussed above, in this study we trained second-order motion discrimination in the parafovea and tested transfer to three tasks at the same location: first-order motion discrimination, static second-order orientation discrimination, and static first-order orientation discrimination, with a separate control group that underwent the same testing battery without training. This design allowed us to test whether learning occurs at the motion extraction stage (Layer e) by examining the pattern of transfer to these tasks.

## Methods

### Subjects

Twenty-four subjects (15 males and 9 females, average age = 23.3 ± 1.5 years) were recruited in this study. All subjects had normal or corrected-to-normal visual acuity. They were randomly assigned into a training group (16) and a control group (8). All subjects were naive to the purpose of the psychophysical experiments. This research has been approved by the ethics committee of the University of Science and Technology of China, and was performed in accordance with the ethical standards laid down in the 1964 Declaration of Helsinki. The written informed consent was obtained from all participants before participation.

Sample size was determined based on previous perceptual learning studies on motion discrimination (Zanker, 1999; Chen et al., 2009; Petrov and Hayes, 2010). A post-hoc sensitivity power analysis (G*Power; Faul et al., 2007) was performed for the training group (*n* = 16), which formed the basis for the primary within-group and correlation analyses. With *α* = 0.05 and power = 0.80, this sample was sufficient to detect a correlation of *r* = 0.50. For the control group (*n* = 8), the minimum detectable correlation under the same criteria was *r* = 0.71, indicating that this group was only powered to detect very large effects. This is consistent with our cautious interpretation of control-group null results in the Limitations.

### Apparatus

The stimuli were displayed on a gamma-corrected 17-inch Sony G220 CRT monitor with a spatial resolution of 640*480 pixels, a refresh rate of 100 Hz, and a mean luminance of 37 cd/m^2^. A special circuit (Li et al., 2003) was used to produce 14 bits of gray levels.

### Visual stimuli and experimental design

The stimuli were generated in real time using Matlab programs based on Psychtoolbox version 2.54 (Brainard, 1997; Pelli, 1997) and were viewed monocularly at a distance of 1.14 m in a dimly lit room. Monocular viewing was adopted to minimize variability arising from interocular differences and binocular rivalry, consistent with the experimental paradigm used in our previous parafoveal perceptual learning study on first- and second-order motion direction discrimination (Chen et al., 2009). This approach also allowed for precise control of retinal eccentricity for the parafoveal stimuli without the additional demands of binocular fusion.

A dual-task paradigm was used to ensure that the training and the measurement were performed in the parafoveal visual field. As shown in Figure 1, subjects were required to perform a central task and a parafoveal task simultaneously. The central task consisted of alphanumeric characters (“S,” “5” and some random numbers) subtending approximately 0.144 × 0.288° of visual angle. Subjects were required to discriminate the “S” from the “5.” The parafoveal task was a static orientation discrimination task or a motion direction discrimination task. Subjects were required to report the orientation, vertical or horizontal, of first- and second-order gratings in the orientation task, and to report the direction, leftward or rightward, of moving first- and second-order stimuli in the motion task. The luminance at point (x, y) of the first- (Equation 1) and second-order (Equation 2) stimuli are defined as:

$$l(x,y)=L_{\text{mean}}\{\begin{array}{l} \;1+R(x,y)C_{C}+C•\sin\\ \left\{2\pi [f(\begin{array}{l} y\cos\theta \\ -x\sin\theta \end{array})+\mathrm{\omega t}]+\phi \right\} \end{array}\}$$ (1)

$$l(x,y)=L_{\text{mean}}\;\{1+R(x,y)C_{c}\;\{\begin{array}{l} 1+C•\sin\\ \left[2\pi (f(\begin{array}{l} y\cos\theta \\ -x\sin\theta \end{array})+\mathrm{\omega t})+\phi \right] \end{array}\}\}$$ (2)

**Figure 1:**
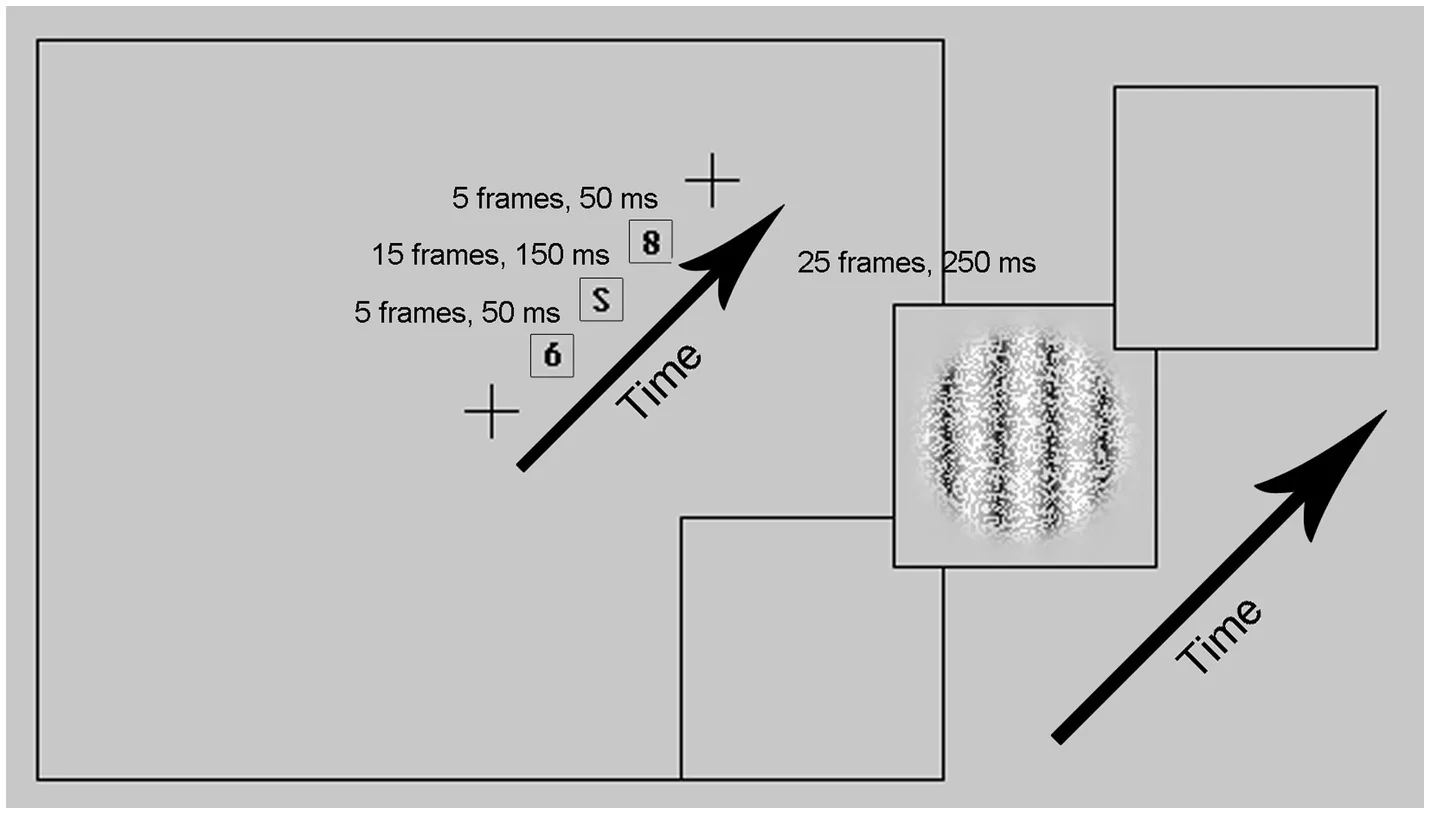
A typical trial sequence for the dual-task paradigm. Subjects were required to perform a central task and a parafoveal task simultaneously. The central task consisted of alphanumeric characters (“S,” “5” and some random numbers) subtending approximately 0.144°x0.288° of visual angle. Subjects were required to discriminate the “S” from the “5.” At the same time, first- or second-order stimuli appeared in the lower-right quadrant. Subjects were required to indicate the orientation, vertical or horizontal, of static first- and second-order gratings in the orientation task, and to indicate the direction, leftward or rightward, of moving first- and second-order stimuli in the motion task.

Where $L_{\text{mean}}$ is the background luminance of the display; R(x,y) is a dynamic noise carrier, consisting of single-pixel dots whose values were randomly assigned as 1 or −1; Cc is the contrast of the noise carrier (here set to 0.5); f is the spatial frequency of the sine wave grating; θ represents the orientation of the grating (0° or 90° in the static orientation task, or 90° in the motion direction discrimination task); ω is the temporal frequency of the moving grating (when ω is set to 0, the stimulus is static); ϕ is the (random) initial spatial phase; and C is the grating contrast. The stimuli in the parafoveal perceptual task were displaced to the lower-right quadrant relative to fixation by 2.3° vertically and 3.1° horizontally.

The experiment consisted of three consecutive phases: pre-training assessment, training, and post-training reassessment. In the pre-training assessment, contrast thresholds for first- and second-order stimuli, both static and moving, were measured for each subject. Two short sessions, each containing 100 trials, were used for the static orientation task. Another two long sessions, each containing 600 trials, were used for motion tasks. Three spatial frequencies (1, 2 and 4 cycles/degree) were used in the static tasks. A fixed spatial frequency (2 cycles/degree) and six different temporal frequencies (2, 4, 8, 14, 21, 30 Hz for first-order motion; 2, 4, 8, 14, 17, 21 Hz for second-order motion) were used in motion tasks. During the experiment, stimuli with different spatial or temporal frequencies were mixed together within trial blocks. The pre-training assessment took a total of 2 h, completed in 2 days. It was followed by 8 days of second-order motion training, 600 trials per day, and 2 days of post-training assessment. A fixed spatial frequency (2 cycles/degree) and a fixed temporal frequency (8 cycles/s) were used in the second-order motion training. The design of the post-training assessment was the same as that of the pre-training assessment.

The training and measurement were only for subjects’ dominant eyes. In other words, subjects’ non-dominant eyes were occluded during experiments. Different training protocols were assigned to the training group and the control group. The training group completed all three phases while the control group only completed the pre- and post-training assessments. In other words, the control group received no training. The pre- and post-training assessments in the control group were separated by 8 days.

By using a two-alternative-forced-choice design and a three-down one-up staircase procedure (Levitt, 1971), a contrast threshold (converging at 79.4% correct) of each subject was measured for each parafoveal perceptual task. Contrast sensitivity (reciprocal of contrast threshold) was used for data analysis.

### Procedure

The display sequence for a typical trial is shown in Figure 1. Following the subject’s keypress, frames for the central task and the parafoveal perceptual task appeared simultaneously. The central task display consisted of a sequence of three letters/numbers with the middle letter either an S or a 5 appearing at the same location as the fixation point. The perceptual task appeared in the lower-right quadrant of the monitor, and consisted of a 250 ms presentation of a first- or second-order stimulus. Trials ended with a fixation display. Then the subject indicated her/his decision with a button press. Auditory feedback was provided in training sessions but not in the pre- and post-training assessments.

### Statistical analysis

All statistical analyses were performed using SPSS 19.0. Repeated-measures ANOVAs were used for within-subject comparisons (training group pre- vs. post-training), with Greenhouse–Geisser correction applied when the sphericity assumption was violated. Mixed-design ANOVAs were used for between-group comparisons (training vs. control), with stimulus type and frequency as within-subject factors and group as a between-subject factor. Partial eta-squared (*η*^2^) is reported as a measure of effect size. Post-hoc comparisons were conducted using paired *t*-tests with Bonferroni correction when applicable. Correlation analyses were performed using Pearson’s correlation coefficient. All tests were two-tailed, and the significance level was set at *α* = 0.05. Data are presented as mean ± 95% CI unless otherwise noted.

To verify the robustness of the mixed ANOVA results to the unequal group sizes (training group, *n* = 16; control group, *n* = 8), we performed supplementary linear mixed-effects models (LMM) using SPSS, with Satterthwaite approximation for degrees of freedom. We first attempted to fit a maximal random-effects structure with random intercepts and random slopes for Time and Frequency by subject: CS ~ Group * Time * Frequency + (1 + Time + Frequency | Subject). This model did not converge, likely due to the complexity of the random-effects structure relative to the sample size (24 subjects × 6 frequency levels). We then simplified the random structure by retaining only the random slope for Time (the within-subject factor with the fewest levels, k = 2): CS ~ Group * Time * Frequency + (1 + Time | Subject). This model also failed to converge. We therefore adopted a random-intercept-only model: CS ~ Group * Time * Frequency + (1 | Subject). Frequency was treated as a categorical factor. Although this model does not fully capture all sources of within-subject variability, it represents the most complex structure that converged with our data and remains a valid robustness check for the unbalanced group sizes. The results were consistent with the ANOVA findings (see Results).

For null results, we performed equivalence testing using the TOST (two one-sided tests) procedure (Lakens, 2017), with equivalence bounds set to Δ = 0.2 for Cohen‘s d and r = ±0.2 for correlations. Bayesian analyses were conducted using JASP (JASP Team, 2024), with Cauchy priors (scale = 0.707), and Bayes factors (BF₀₁) were interpreted following Jeffreys (1961).

Sensitivity power analyses were performed using G*Power (Faul et al., 2007; *α* = 0.05, power = 0.80).

## Results

### Central task performance

Similar to previous studies (Dosher and Lu, 1999; Chen et al., 2009), subjects performed well on the central task (93.9% ± 4.8% across all 24 subjects), indicating that they maintained central fixation throughout the experiment. This confirms that the training and testing stimuli were presented in the parafoveal visual field. Additionally, central task performance did not change systematically before versus after training (paired *t*-test, *t*(23) = 1.658, *p* = 0.111, Cohen‘s d = 0.69), ruling out a tradeoff between accuracy on the central and parafoveal tasks as a result of practice.

### Group comparisons

Results for the training group on the parafoveal perceptual tasks are presented in Figures 2, 3. The learning curve (Figure 2) showed substantial improvement (*F*(1,8) = 101.795, *p* < 0.001, partial *η*^2^ = 0.927), with a slope of 2.01 dB per log unit of training session.

**Figure 2:**
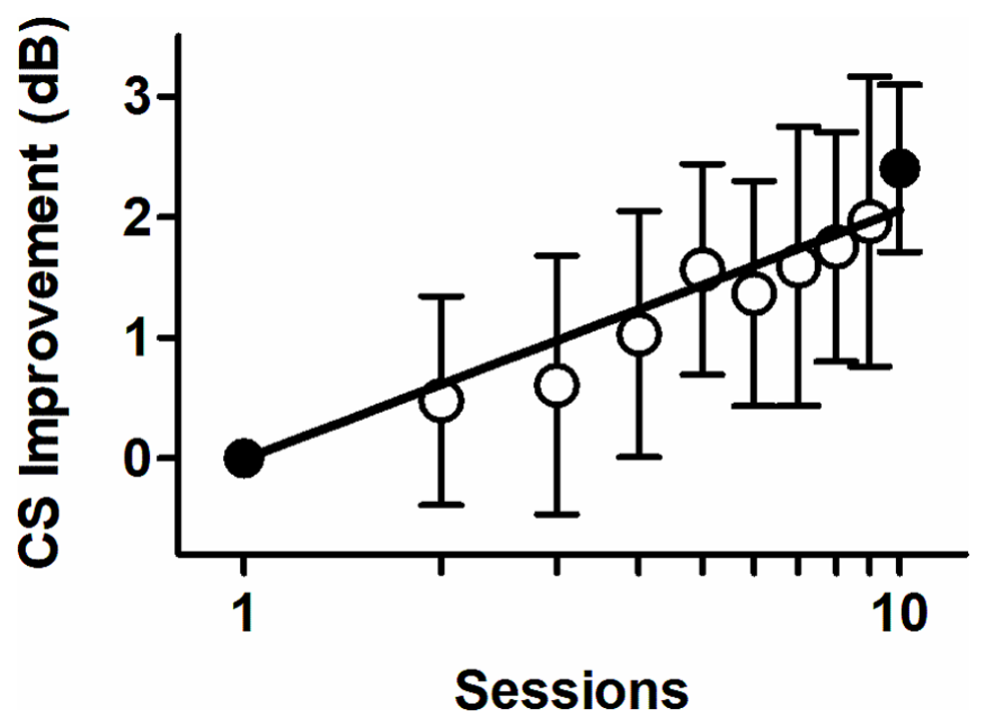
Average learning curve of the training group. The first and last data points (filled circles) were derived from pre-training and post-training tests, respectively. Data from the training phase are represented by open circles. Data were fit with a linear function with a slope of 2.01 and *R*^2^ of 0.97 (*p* < 0.001). Error bars indicate 95% CI.

**Figure 3:**
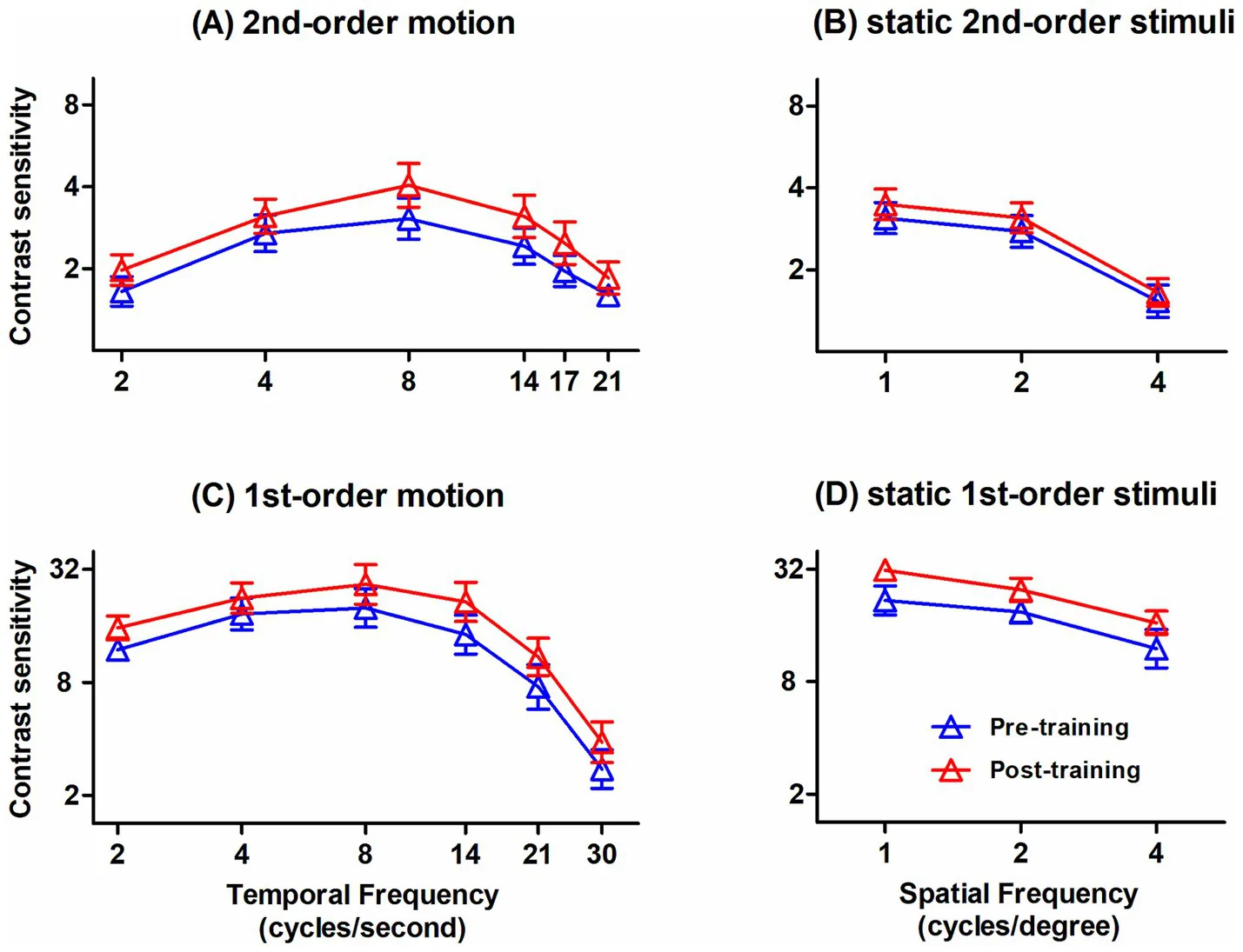
Pre- and post-training contrast sensitivity for first- and second-order stimuli in the training group. Significant improvements in perception of second-order motion **(A)**, static second-order stimuli **(B)**, first-order motion **(C)**, and static first-order stimuli **(D)** were found after training (all *p*s < 0.05). Error bars indicate 95% CI.

Consistent with this, contrast sensitivity for second-order motion improved from pre- to post-training across all six temporal frequencies (Figure 3A), with mean improvements of 1.55 ± 0.43, 1.25 ± 0.37, 2.44 ± 0.26, 2.19 ± 0.40, 2.06 ± 0.37, and 1.25 ± 0.34 dB at 2, 4, 8, 14, 17, and 21 Hz, respectively. Static second-order stimuli also yielded modest but measurable improvements (Figure 3B), with mean improvements of 1.02 ± 0.43, 0.99 ± 0.66, and 0.62 ± 0.55 dB at spatial frequencies of 1, 2, and 4 cycles/degree, respectively. For first-order stimuli, improvements were also prominent (Figures 3C,D). First-order motion showed mean improvements of 2.33 ± 0.37, 1.69 ± 0.68, 2.49 ± 0.51, 3.47 ± 0.38, 3.17 ± 0.64, and 2.87 ± 0.65 dB at 2, 4, 8, 14, 21, and 30 Hz, respectively. Static first-order stimuli showed improvements of 3.24 ± 0.87, 2.38 ± 0.80, and 2.76 ± 1.15 dB at 1, 2, and 4 cycles/degree.

Two-way repeated-measures ANOVAs (time × frequency) confirmed significant main effects of training for both first-order motion (*F*(1,15) = 79.648, *p* < 0.001, partial *η^2^* = 0.842) and second-order motion (*F*(1,15) = 122.068, *p* < 0.001, partial *η^2^* = 0.891). Similar effects were observed for static first-order (*F*(1,15) = 19.571, *p* < 0.001, partial *η^2^* = 0.566) and second-order stimuli (*F*(1,15) = 6.055, *p* = 0.026, partial *η^2^* = 0.288).

Although the training group showed substantial improvements across all conditions, it remains possible that these gains stemmed in part from test–retest practice effects rather than exclusively reflecting the effects of second-order motion training. Results from the control group helped clarify this issue. As shown in Figure 4A, no significant improvement in second-order motion perception was observed between the first and second tests in the control group (*F*(1,7) = 3.905, *p* = 0.088, partial *η^2^* = 0.438). Similarly, no significant improvements were found for static second-order perception (*F*(1,7) = 5.195, *p* = 0.057, partial *η^2^* = 0.426; see Figure 4B) or first-order motion (*F*(1,7) = 3.567, *p* = 0.101, partial *η^2^* = 0.338; see Figure 4C). In contrast, contrast sensitivity for static first-order stimuli increased significantly (*F*(1,7) = 20.554, *p* = 0.003, partial *η^2^* = 0.746; see Figure 4D), with mean gains of 3.11 ± 1.05, 2.55 ± 0.55, and 0.97 ± 0.64 dB for spatial frequencies of 1, 2, and 4 cycles/degree. These findings do not support a practice-effect account of the training-induced gains, as the control group showed no significant improvements in either motion or static second-order tasks, with the only practice effect restricted to static first-order stimuli, the simplest condition tested.

**Figure 4:**
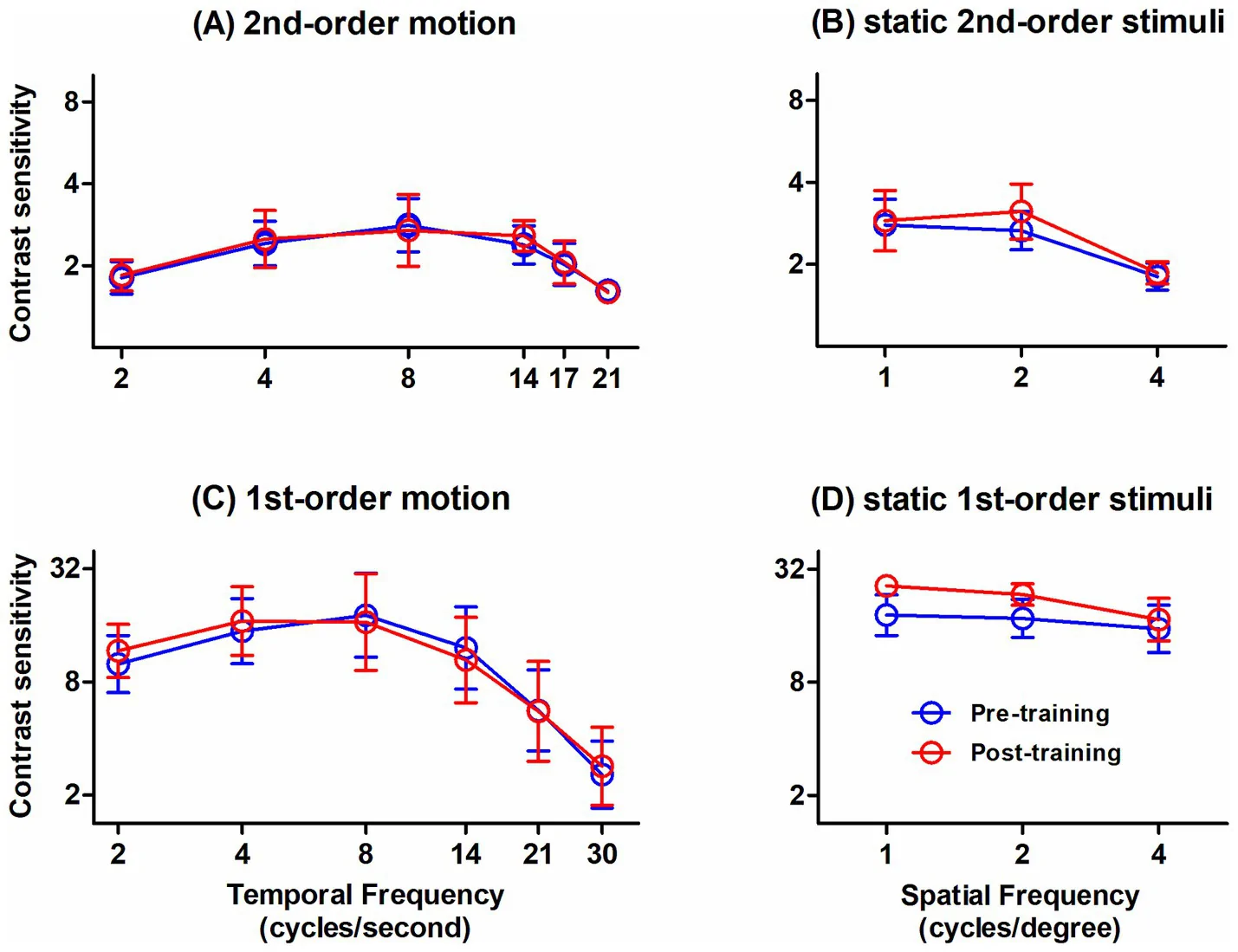
Test and retest contrast sensitivity for first- and second-order stimuli in the control group. Subjects exhibited comparable performance (all *p*s > 0.05) between pre- and post-training tests for second-order motion **(A)**, static second-order stimuli **(B)**, and first-order motion **(C)**. In contrast, a significant improvement (*p* = 0.003) was found in perception of static first-order stimuli **(D)**. Error bars indicate 95% CI.

We next compared improvements between the training and control groups. A three-way mixed-design ANOVA (stimulus type × spatial frequency × group) was conducted for static stimuli. Results revealed a significant main effect of stimulus type (*F*(1,22) = 11.346, *p* = 0.003, partial *η^2^* = 0.340), indicating that improvements were greater for static first-order than second-order stimuli. However, the main effect of group and all group-related interactions were not significant (all *ps* > 0.5), consistent with the interpretation that improvements in static stimulus perception were comparable between the two groups.

A separate three-way mixed-design ANOVA (motion type × temporal frequency × group) was conducted for motion stimuli. Results revealed a significant main effect of group (*F*(1,22) = 19.139, *p* < 0.001, partial *η^2^* = 0.465), with no significant group-related interactions (all *ps* > 0.25). This indicates that the training group showed significantly greater improvements in motion perception than the control group, consistent with a specific effect of second-order motion training.

To address the potential concern regarding the unbalanced group sizes (16 vs. 8) in the mixed ANOVA, we performed a supplementary analysis using linear mixed-effects models (LMM) with Satterthwaite approximation for degrees of freedom. The model included Group, Time, and Frequency as fixed factors with all interactions, and subject as a random intercept. The analysis was conducted on the full dataset (24 subjects × 2 time points × 6 temporal frequencies = 288 observations).

The LMM results confirmed the ANOVA findings. The Group × Time interaction was highly significant (*F*(1, 242) = 17.487, *p* < 0.001, partial *η*^2^ = 0.067), indicating that the training group showed significantly greater improvements in second-order motion perception than the control group. The three-way interaction Group × Time × Frequency was not significant (*F*(5, 242) = 0.394, *p* = 0.853, partial *η*^2^ = 0.008), indicating that this training-specific improvement did not depend on temporal frequency. These results confirm that the primary conclusion is robust to the unbalanced design.

Together, these results suggest that second-order motion training preferentially enhanced motion perception, as indicated by the significant group differences for motion stimuli. In contrast, although improvements in static stimulus perception were significant within the training group, they were comparable between the training and control groups, suggesting that these improvements were not specific to the training.

### Relationships among improvements

The improvements averaged across temporal frequencies for first- and second-order motion are shown in Figure 5, and those averaged across spatial frequencies for static first- and second-order stimuli are shown in Figure 6. Linear fitting revealed no significant dependence on spatial or temporal frequency (all *ps* > 0.05). This conclusion was further supported by ANOVA results, which showed no significant main effects or interactions involving spatial or temporal frequency (all *ps* > 0.05), indicating general improvements across these frequency dimensions.

**Figure 5:**
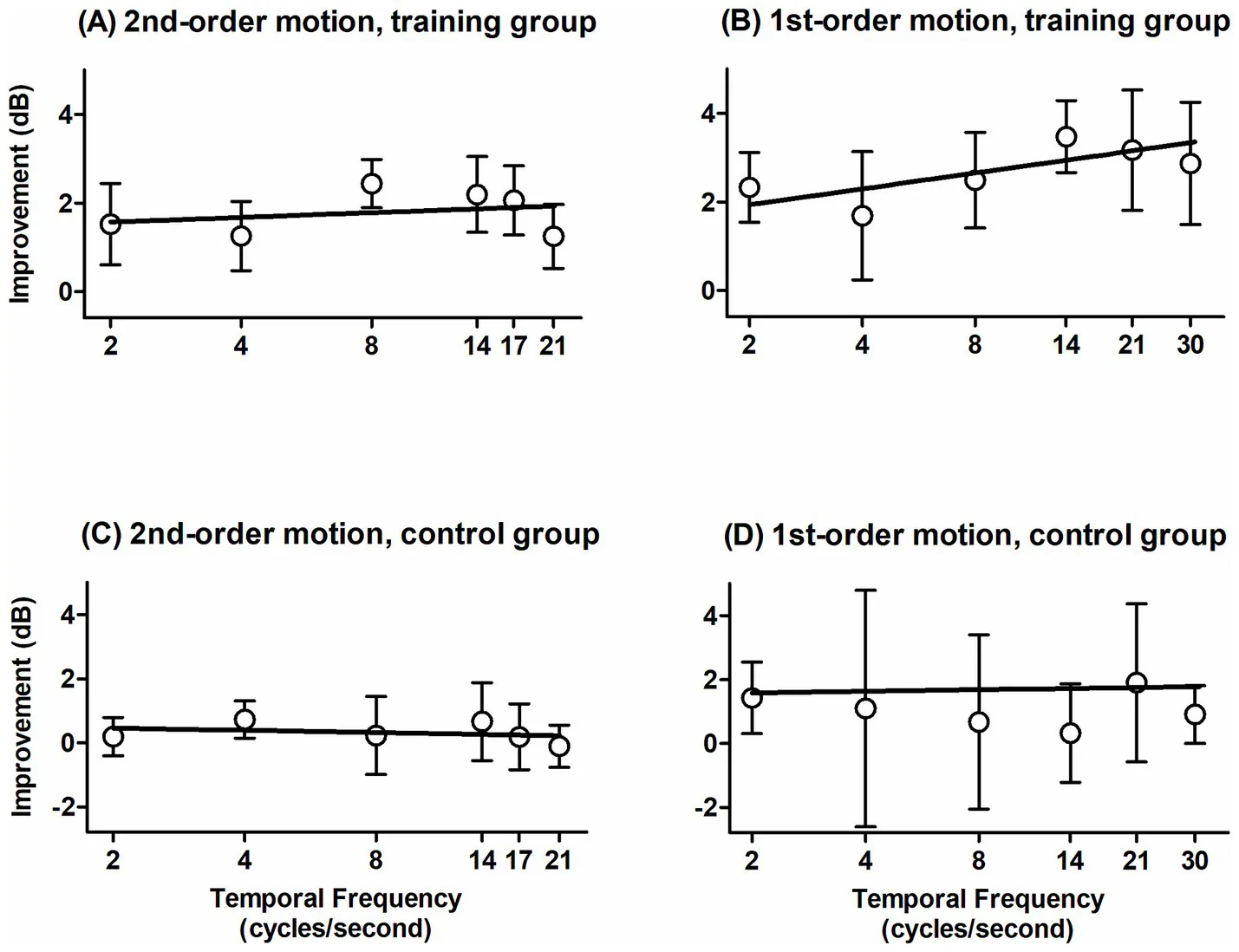
Improvements of contrast sensitivity for second-order motion in the training group **(A)**, first-order motion in the training group **(B)**, second-order motion in the control group **(C)**, and first-order motion in the control group **(D)**. Significant differences were found in improvements between two groups (*F*(1, 22) = 19.139, *p* < 0.001) and between two types of motion (*F*(1, 22) = 9.791, *p* = 0.005). In each case, improvements did not depend on the temporal frequency (linear fitting, *p* = 0.596, 0.569, 0.092, and 0.738, respectively). Error bars indicate 95% *CI*.

**Figure 6:**
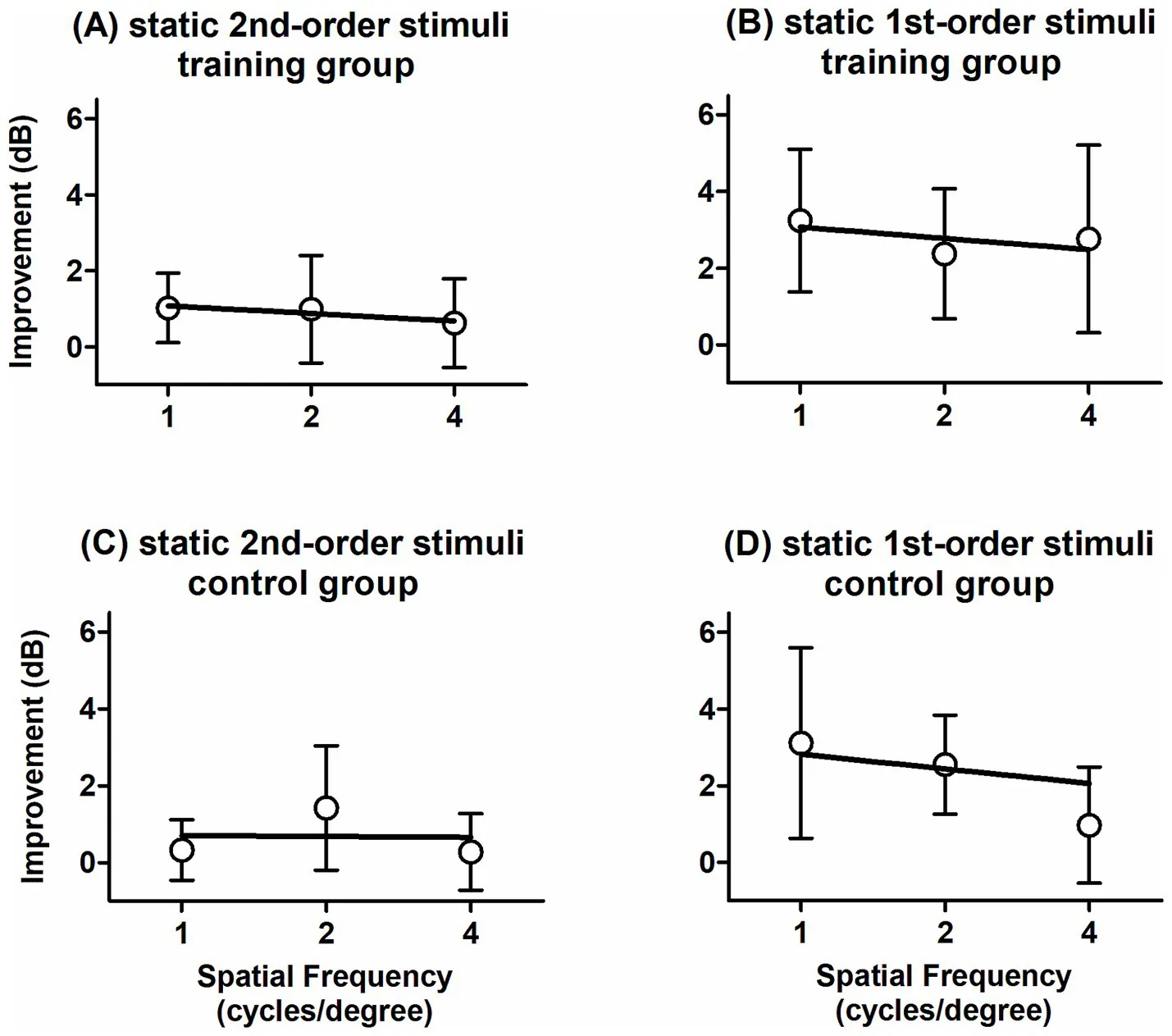
Improvements of contrast sensitivity for static second-order stimuli in the training group **(A)**, static first-order stimuli in the training group **(B)**, static second-order stimuli in the control group **(C)**, and static first-order stimuli in the control group **(D)**. Improvements were significantly greater for first-order stimuli than for second-order stimuli (*F*(1,22) = 11.346, *p* = 0.003), but were similar (*F*(1,22) = 0.283, *p* = 0.600) between training and control groups. In each case, improvements did not depend on the temporal frequency (linear fitting, *p* = 0.287, 0.978, 0.111 and 0.831, respectively). Error bars indicate 95% CI.

Notably, first-order stimuli exhibited greater improvements than second-order stimuli, for both static (*F*(1,22) = 11.346, *p* = 0.003, partial *η^2^* = 0.340) and motion (*F*(1,22) = 9.791, *p* = 0.005, partial *η^2^* = 0.308) conditions. However, it would be premature to conclude that first-order motion benefited more from training than second-order motion, because greater improvements in first-order stimuli were observed in both the training and control groups. Indeed, the same ANOVA did not reveal a significant two-way interaction between motion type and group (*F*(1,22) = 0.006, *p* = 0.940, partial *η^2^* < 0.001) or a three-way interaction (*F*(2,21) = 0.588, *p* = 0.564, partial *η^2^* = 0.053). This pattern is consistent with the interpretation that the greater improvements for first-order motion reflected a general stimulus-type difference rather than a training-specific effect, although the statistical power to detect such interactions was limited.

Based on the findings described above, we conducted further correlation analyses to examine the relationships among improvements in perception, averaged across spatial and temporal frequencies, for second-order motion, first-order motion, and static first- and second-order stimuli. As shown in Figure 7A, there was a significant correlation between improvements in CS for first- and second-order motion (*F*(1,14) = 7.786, *p* = 0.014, Pearson *r* = 0.598), with a slope of 1.10 ± 0.39. In contrast, no significant correlation was found between improvements in second-order motion perception and those in static second-order perception (Figure 7B; *F*(1,14) = 0.994, *p* = 0.336, Pearson *r* = −0.063). Additionally, improvements in static first-order perception did not correlate with those in first-order motion (Figure 7C; *F*(1,14) = 0.685, *p* = 0.422, Pearson *r* = −0.062) or first-order motion (Figure 7D; *F*(1,14) = 0.646, *p* = 0.435, Pearson *r* = −0.381).

**Figure 7:**
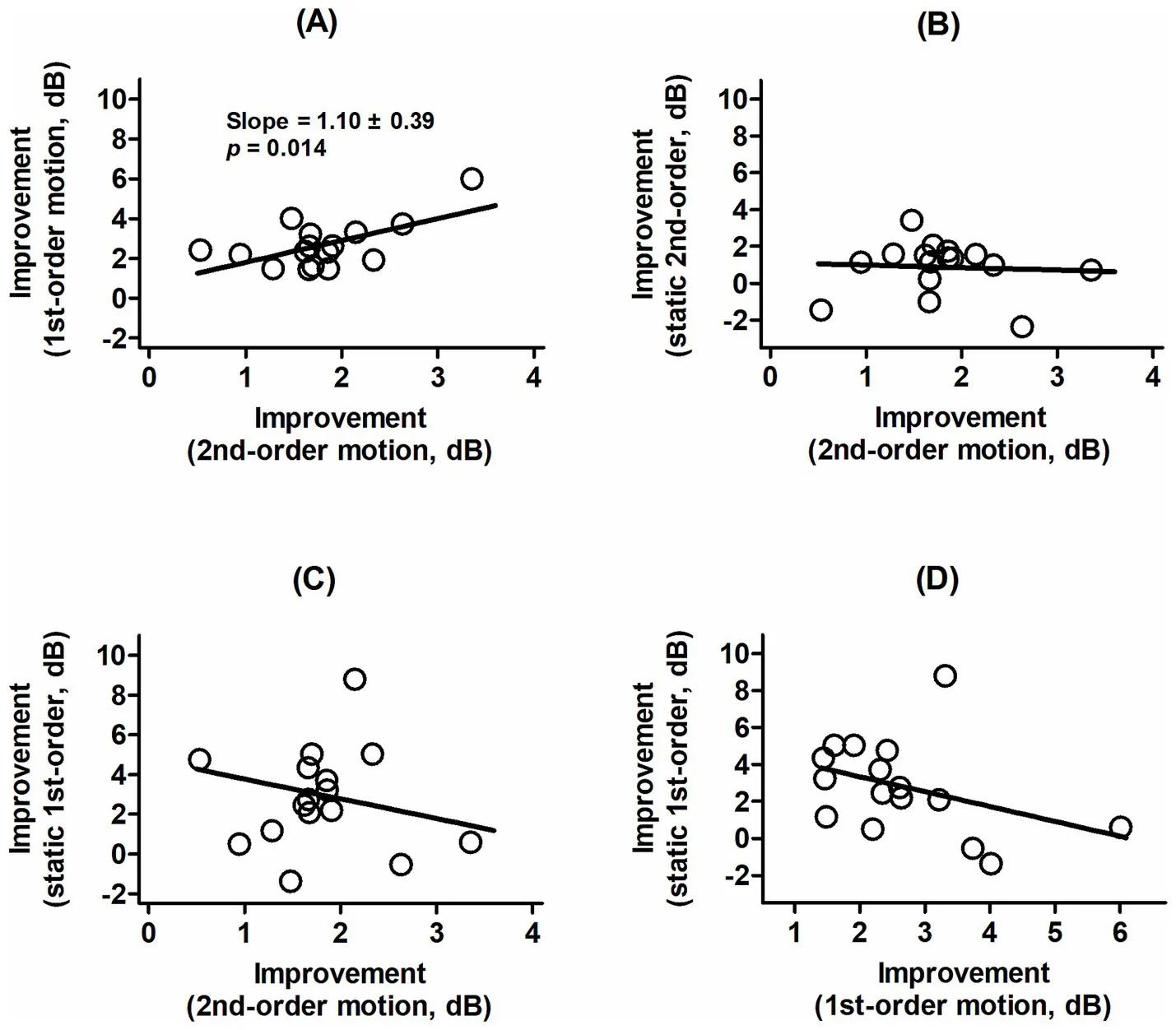
Relationships between improvements in first- and second-order stimulus performance in the training group. CS for first- and second-order motion (averaged across spatial/temporal frequencies) showed a significant correlation **(A)**, whereas CS for second-order motion and static second-order stimuli did not **(B)**. Improvements in CS for second-order **(C)** and first-order **(D)** motion also showed no significant correlations with improvements in static first-order perception.

The significant group difference in motion improvement provides direct evidence that the training had a specific effect on motion perception. The correlation between improvements in first- and second-order motion (slope: 1.10 ± 0.39) further supports substantial cross-transfer between these motion types. Together, these findings suggest that second-order motion training primarily enhanced motion-related mechanisms, with no reliable evidence of specific effects on static structure processing.

### Equivalence testing

To directly assess whether the lack of significant transfer to static second-order perception reflects a genuine absence of effect or insufficient power, we performed equivalence tests and Bayesian analyses on two critical null comparisons (see Methods for details).

For the group comparison in static second-order improvements, TOST with equivalence bounds set to Δ = 0.2 (Cohen‘s d) did not reach formal statistical equivalence (one of the two one-sided tests was not significant), largely due to the small control group sample size (*n* = 8). The group difference was small (0.34 dB), and the Bayesian independent-samples t-test yielded BF₀₁ = 1.85. Following Jeffreys (1961) classification, this BF₀₁ = 1.85 falls in the anecdotal range (1–3), indicating that the data are only weakly more likely under the null than under the alternative hypothesis. On its own, this group-comparison result should therefore be interpreted as inconclusive.

For the correlation between second-order motion and static second-order improvements (Pearson *r* = −0.063, *n* = 16), the Bayesian correlation test yielded BF₀₁ = 3.07, which falls at the low end of the moderate evidence range (3–10) for the null hypothesis (Jeffreys, 1961), indicating that the data are three times more likely to support the absence of correlation than the presence of one.

Thus, while strict statistical equivalence was not achieved in the TOST framework for the group comparison, the combined evidence from the two Bayesian analyses—anecdotal evidence for the group comparison and moderate evidence for the correlation—suggests that any transfer effect to static second-order perception, if present, is likely to be small. However, given the inconclusive nature of the group-comparison result, we emphasize that future studies with larger samples are needed to provide a more definitive test of transfer to static second-order perception.

### Robustness checks

In the comparison described above, a potential concern arises regarding stimulus orientation. For the assessment of static second-order stimuli, patterns with two orthogonal orientations (vertical and horizontal) were used, and both contributed equally to the measured contrast sensitivity (see Methods). In contrast, only vertically oriented patterns were employed in the second-order motion training task. Thus, training likely improved perception of vertically oriented patterns, but not necessarily of horizontally oriented ones. Consequently, training-induced gains in static second-order perception could be underestimated—by as much as 50%—if training produced no benefit for horizontally oriented patterns.

To determine whether this potential underestimation affected our conclusions, we performed a re-analysis in which the static contrast sensitivity gains for the training group were artificially inflated by 100% (i.e., doubling the observed improvement, corresponding to the worst-case scenario in which horizontal-orientation gains were completely absent). The main results are shown in Figure 8. Improvements in static second-order perception remained comparable between the training and control groups (*F*(1,22) = 1.370, *p* = 0.254, partial *η^2^* = 0.059; see Figure 8A), and no significant correlation emerged between improvements in static second-order perception and those in second-order motion within the training group (*F*(1,14) = 0.994, *p* = 0.336, Pearson *r* = −0.063; see Figure 8B). Together, these findings suggest that our original conclusions are unlikely to be compromised by the orientation imbalance, although this analysis has inherent limitations and a more direct test (e.g., an ANOVA including orientation as a factor) would be desirable.

**Figure 8:**
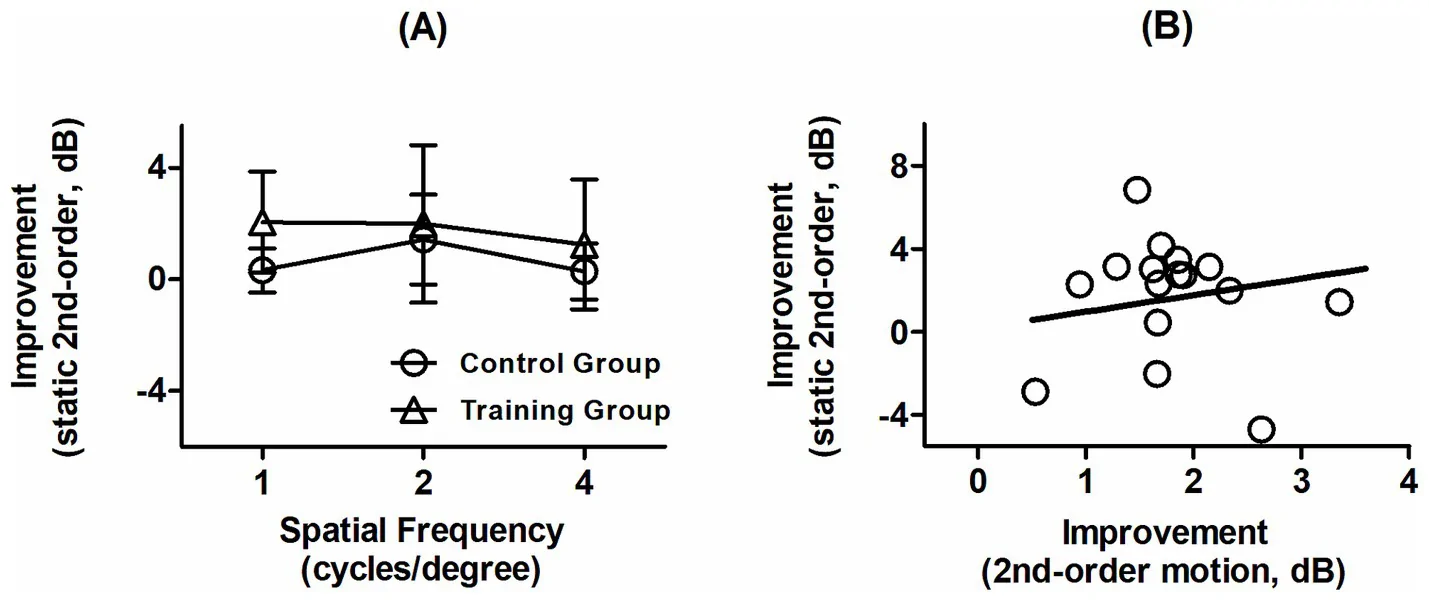
Re-analysis after inflating training-group gains in static second-order perception by 100%. The re-analyzed data still showed no significant group difference between the training and control groups **(A)** and no significant correlations between improvements in second-order motion and static second-order perception within the training group **(B)**. Error bars indicate 95% CI.

We also examined whether the significant correlation between first- and second-order motion improvements (Figure 7A; Pearson *r* = 0.598, *p* = 0.014) was driven by the rightmost data point. After excluding this participant, the correlation was no longer significant (Pearson *r* = 0.234, *p* = 0.201, *n* = 15), suggesting that this particular correlation should be interpreted with caution.

## Discussion

We trained second-order motion discrimination in the parafovea and tested for transfer to first-order motion and static orientation discriminations. Three results stand out. Training improved contrast sensitivity for both second-order and first-order motion, with roughly comparable gains between the two. Static second-order perception, by contrast, improved about as much in the control group as in the training group. And in the training group, the improvements in first- and second-order motion were correlated (slope ≈ 1.10), while no such correlation appeared between second-order motion and static second-order perception. Taken together, these results point to a training effect that is largely motion-specific, with no reliable evidence of transfer to static structure processing.

### Comparison with previous studies on motion perception

As described in the Introduction, several previous studies examined the transfer of perceptual learning from second-order to first-order motion, both in central (Zanker, 1999; Petrov and Hayes, 2010) and parafoveal (Chen et al., 2009) visual fields. Although there was a large variation of training durations in these studies, they all showed significant improvements in second-order motion perception. After about 800 trials’ training, Zanker found that subjects could perform the task as well as before even though the noise superimposed on the motion stimuli was increased by 10%. In Petrov and Hayes’ study, the motion direction discriminability improved almost two-fold after 3,328 trials’ training. And in Chen et al.’s study, contrast sensitivity for second-order motion in parafoveal visual field improved about 1.98 dB after 4,200 trials’ training. Here, we found substantial training-induced improvements in contrast sensitivity for second-order motion, about 1.8 dB averaged across subjects and temporal frequencies, consistent with all the findings described above, especially those in Chen et al.’s study.

Another related study should be mentioned (Vaina and Chubb, 2012). Vaina and Chubb reported a double dissociation between first- and second-order motion systems using a perceptual learning paradigm. They found that training on luminance-defined (LD) motion did not transfer to contrast-defined (CD) motion, and training on CD motion did not transfer to LD motion, indicating a pattern that differs from the asymmetric transfer we and others have observed. However, Vaina and Chubb used global motion stimuli, whereas our task and those of Zanker (1999) and Chen et al. (2009) used grating-based direction discrimination. Therefore, this discrepancy likely reflects the different task types employed.

Before leaving this section, we note that the perception of first-order motion appeared to be less sensitive to perceptual learning than that of static first-order stimuli, as indicated by the much larger test–retest improvements for the latter in the control group. Although no direct comparison has been reported previously, two studies on perceptual learning of static and moving first-order stimuli in the central visual field offer relevant evidence. Dosher and Lu (2006) found perceptual learning was small or absent for static first-order orientation identification, whereas Lu et al. (2006) found substantial learning for first-order motion discrimination in all subjects. Given that all subjects in both studies were psychophysically naive, this contrast may reflect a greater degree of real-life experience with static first-order stimuli than with first-order motion.

### Non-specific improvements in static stimulus perception

In the present study, substantial improvements in perception of static first-order stimuli were found for both training and control groups. These improvements were comparable between the two groups, with mean improvements of 2.21 dB for the control group and 2.79 dB for the training group (averaged across subjects and spatial frequencies), suggesting that they were not specific to the motion training but rather reflected rapid learning induced by repeated testing. Such rapid learning has been reported previously in various tasks (Fiorentini and Berardi, 1980; Karni and Sagi, 1993; Dosher and Lu, 1999). This rapid learning effect may be particularly pronounced in the parafovea, where baseline performance is lower and thus greater room for improvement exists (Levi, 2008). Consistent with this notion, previous studies have shown that training simple, low-level tasks at the fovea often yields little or no perceptual learning (Fiorentini and Berardi, 1981; Dorais and Sagi, 1997; Matthews et al., 1999; Furmanski and Engel, 2000), whereas comparable tasks in the parafovea can produce substantial gains (Dosher and Lu, 1998; Dosher and Lu, 1999).

A central aim of this study was to determine whether second-order motion training improves perception of static second-order stimuli, as this bears directly on the locus of learning. Only subtle changes in contrast sensitivity were observed for static second-order stimuli in the training group, and these improvements were not significantly different from those in the control group, even under a conservative re-analysis that doubled the training group’s gains (Figures 6, 8). These findings suggest that the improvements in static second-order perception arose from pre- and post-training test familiarity rather than from the motion training itself.

Further evidence supporting this interpretation comes from the observation that improvements in static stimulus perception, for both first-order and second-order stimuli, were independent of spatial frequency. The pre- and post-training tests included multiple spatial frequencies, whereas training used only a single frequency (2 c/deg). Given that static processing, for both first- and second-order stimuli, is typically selective for spatial attributes such as orientation (Mareschal and Baker, 1998; Landy and Graham, 2004; Larsson et al., 2006; Li et al., 2013) and spatial frequency (Baker and Mareschal, 2001; Hallum et al., 2011; Li et al., 2013), the spatial-frequency independence of the observed improvements is more consistent with general test familiarity than with training-induced plasticity.

A related point concerns the differential sensitivity of static first- and second-order stimuli to perceptual learning. The improvements for static first-order stimuli were substantially larger than those for static second-order stimuli, even though both appear to reflect non-specific test–retest effects. This difference may reflect their different status in everyday visual experience: humans likely have extensive real-life exposure to static first-order patterns, whereas static second-order patterns are encountered less frequently. Consistent with this, Dosher and Lu found that five sessions of training produced systematic improvements in second-order perception but little in first-order perception, suggesting that first-order static processing may already be near its performance ceiling due to extensive real-life exposure (Dosher and Lu, 2006). While this account is plausible, our present data are not sufficient to fully clarify this issue, and further investigation is warranted.

### Locus of learning

To date, proposed systems for the processing of static second-order stimuli (Wilson et al., 1992; Sutter and Graham, 1995) share formal properties with the systems for processing second-order motion (Chubb and Sperling, 1989; Lu and Sperling, 2001), although perceptual performance with these two stimulus classes can dissociate in aging (Tang and Zhou, 2009) and amblyopic populations (Tang et al., 2014). Given this similarity, one might have expected that training on second-order motion would improve perception of static second-order stimuli as well, if the two types of stimuli relied on shared processing mechanisms. However, this was not the case. To understand why, consider the two-stage theory of motion perception proposed by Albright (Albright and Stoner, 1995), according to which the first stage extracts image features, such as second-order structure, and the second stage “matches” these features in space–time, i.e., motion information processing. If the changes induced by training had occurred at the first stage, we would have expected comparable improvements for both motion and static second-order stimuli, because both rely on similar texture-defined inputs processed at this stage. The fact that specific improvements, those exceeding non-specific practice effects, were largely confined to motion stimuli thus points to a later stage, specialized for motion processing, a conclusion consistent with the two-stage framework and with our hypothesis that learning occurs at the motion extraction stage (Layer e) of the non-Fourier pathway.

The investigation of changes in first-order motion perception provided another insight into this issue. Contrast sensitivity for first-order motion improved in the training group but not in the control group, unlike for static first-order stimuli. These results suggest that the improvements in first-order motion perception resulted from a transfer effect of second-order motion training. Further data analyses indicated a near-complete transfer from second- to first-order motion, with the slope of the correlation between improvements in the two tasks (1.10 ± 0.39) not significantly different from unity (*p* > 0.05). This suggests that the neural substrates underlying the plasticity site during second-order motion training are form-cue invariant. These findings are consistent with three previous studies (Zanker, 1999; Chen et al., 2009; Petrov and Hayes, 2010) and provide strong support for the notion that first- and second-order motion share a common pathway at the motion extraction stage.

This view of shared processing within the non-Fourier pathway is broadly consistent with observations from the motion aftereffect literature (Schofield et al., 2007). Schofield and his colleagues demonstrated an asymmetric transfer of the dynamic motion aftereffect between first- and second-order motion cues. Specifically, they found that adaptation to luminance-modulated (first-order) motion produced a partial aftereffect on contrast-modulated (second-order) motion, a transfer that lost its spatial-frequency tuning. This loss of tuning suggests the involvement of a later, more abstract stage of motion processing, which resonates with our proposal of a shared, form-cue invariant plasticity site at the motion extraction stage (Layer e) for second-order motion learning. The partial nature of the transfer in that study, as opposed to the near-complete transfer we observed from second- to first-order motion, is consistent with the asymmetric architecture of the dual-pathway model (Petrov and Hayes, 2010), in which the non-Fourier pathway responds to both cues whereas the Fourier pathway responds only to luminance-defined signals. This architecture predicts that learning-induced strengthening of the non-Fourier pathway (as in our second-order training) would yield near-complete transfer to first-order motion, whereas adaptation effects that rely more heavily on the Fourier pathway (as in first-order adaptation) would yield only partial transfer to second-order motion, an asymmetry that aligns well with both Schofield et al.’s findings and our own.

This pattern of results can be understood within the reweighting framework of perceptual learning (Lu and Dosher, 2022). According to this framework, learning optimizes the connection weights between sensory representations and perceptual decisions rather than altering the early representations themselves. Applied to our findings, second-order motion training appears to have selectively strengthened the connections from the shared motion extraction stage (Layer e) to a common decision unit for motion direction, without affecting connections that support static perceptual decisions. This would explain why improvements transferred to first-order motion (which shares this stage) but not to static second-order perception (which does not engage it), and why improvements in first- and second-order motion were correlated whereas those in second-order motion and static second-order were not.

Taken together, our findings suggest that the plasticity site during second-order motion training is located at a late stage of the second-order processing system, where underlying neuronal substrates conduct motion information processing and exhibit form-cue invariance. In view of this, area MT + may be a good candidate for this site, since neurons in this area exhibit marked motion directional selectivity with a conspicuous absence of selectivity for form or color (Zeki, 1974; Maunsell and van Essen, 1983; Albright, 1984). However, it is also possible that this site is located earlier in the visual hierarchy than MT+, potentially as early as V1. Although V1 has traditionally been considered a primary area for processing first-order static features, recent evidence demonstrates that V1 superficial-layer neurons respond substantially to second-order contrast-modulated stimuli, with orientation tuning potentially arising from a subcortical-to-V1 filter-rectify-filter mechanism (Ju et al., 2022). This raises the possibility that the locus of learning-induced plasticity may reside as early as V1. Alternatively, the site could be located at intermediate levels, such as V2, where some neurons have been shown to be selective to both first- and second-order motion directions (Zhou and Baker, 1993; Zhou and Baker, 1996; Li et al., 2013). Overall, more studies are still needed to clarify the precise neural locus of this learning.

### Limitations

Several limitations should be acknowledged.

First, the motion and static tasks inherently differed in their stimulus attributes, direction discrimination for motion and orientation discrimination for static. This difference is unavoidable, as static stimuli lack a motion trajectory. However, it is important to note that both tasks required only coarse, categorical discriminations: motion direction differed by 180° (left vs. right) and orientation differed by 90° (vertical vs. horizontal). Previous studies have shown that such coarse discriminations are essentially equivalent to detection thresholds (Thomas and Gille, 1979; Qiu et al., 2007). For example, direction discrimination thresholds for 180°-separated motions are statistically indistinguishable from motion detection thresholds in normal controls and most amblyopic patients (Qiu et al., 2007), and coarse orientation discrimination thresholds are similarly comparable to orientation detection thresholds (Thomas and Gille, 1979). Thus, although the specific labels differ, the underlying task structure, a binary categorical decision at a detection-like level, is functionally comparable across tasks.

Second, training used only vertically oriented stimuli, whereas the static assessment included both vertical and horizontal orientations. This asymmetry raises the concern that training-induced improvements may have been diluted by the inclusion of horizontal orientations. However, we emphasize that this was not an arbitrary design choice but a logical requirement of the discrimination paradigm. To measure orientation discrimination (rather than mere detection), the task must include two alternative categories (e.g., vertical vs. horizontal). A static test using only vertical orientation would have reduced the task to detection (‘is the grating present or absent?’), which would no longer serve as a proper control for the motion discrimination task. Thus, the inclusion of both orientations was essential to maintain the discrimination-demand structure. We attempted to address the orientation asymmetry with a conservative re-analysis in which we artificially doubled the training group’s static second-order gains (assuming zero transfer to horizontal orientations; see Figure 8). Even under this worst-case scenario, the training and control groups still showed no significant difference in static second-order improvements, confirming that the conclusion is robust to the orientation imbalance. Nevertheless, we acknowledge that a direct measurement of vertical-only static thresholds would have been a superior control, and we note this as an important direction for future research. It should also be noted that the post-hoc 100% inflation analysis presented in Figure 8 is a crude approximation and should be interpreted with caution, as it assumes zero transfer to horizontal orientations without directly measuring vertical-only thresholds. Future studies that measure static thresholds separately for each orientation would provide a more definitive test.

Third, we did not include a first-order motion training group, which would have allowed us to replicate the full asymmetry within our own design. While this asymmetry is well established in the literature (Zanker, 1999; Chen et al., 2009; Petrov and Hayes, 2010), and our primary focus was on the static second-order question, including such a group would have further strengthened the study.

Fourth, the control group sample size is small (*N* = 8), which limits power, especially for detecting group-by-condition interactions. Our null results should therefore be interpreted with caution. Consistent with this, several control-group comparisons showed moderate-to-large effect sizes (partial *η*^2^ = 0.338–0.438) that nevertheless failed to reach statistical significance (*p*s = 0.057–0.101), reflecting the limited power inherent to this small sample rather than any inconsistency in the data. However, a conservative re-analysis in which the training group’s static second-order gains were artificially inflated by 100% still yielded no significant group differences (Figures 6, 8), suggesting that the lack of specific transfer is unlikely to be entirely attributable to insufficient power. Importantly, the primary conclusion of this study, that second-order motion training specifically enhances motion processing, rests on the significant group difference in motion improvements (*F*(1,22) = 19.139, *p* < 0.001, partial *η*^2^ = 0.465), which is based on the full sample (n = 24) and is therefore not undermined by the control group’s sample size.

## Conclusion

Second-order motion training in the parafovea improved first-order motion performance substantially, with substantial cross-transfer between first- and second-order motion. By contrast, we found no reliable improvement in static second-order perception beyond practice effects: the training-versus-control group difference was small, and Bayesian analyses provided anecdotal-to-moderate evidence favoring the absence of a meaningful correlation between second-order motion and static second-order improvements. This pattern is consistent with a learning site at the motion extraction stage (Layer e) within the non-Fourier pathway, which processes both first- and second-order signals, rather than at early texture-feature extraction stages (layers b–d). These findings provide behavioral constraints for models of motion perception and perceptual learning, and suggest that second-order mechanisms may be dissociable according to stimulus motion status, despite sharing a common non-Fourier pathway. Future work comparing foveal and parafoveal training, combined with neural measures, would help clarify the generality of these mechanisms.

## Data Availability

The raw data supporting the conclusions of this article will be made available by the authors, without undue reservation.
